# Double-layered fiber for lightweight flexible clothing providing shielding from low-dose natural radiation

**DOI:** 10.1038/s41598-021-83272-3

**Published:** 2021-02-11

**Authors:** Seon-Chil Kim, Jun Sik Son

**Affiliations:** 1 Department of Biomedical Engineering, Keimyung University School of Medicine, Daegu, Korea; 2 Korea Textile Development Institute, Daegu, Korea

**Keywords:** Environmental sciences, Health occupations, Materials science

## Abstract

Natural and medical radiation are the most frequent sources of daily low-dose radiation exposure for the general public, but these radiation levels are generally acceptable. Among various occupations, aviation crew members and medical workers are exposed to high levels of radiation from scattered rays. This study focused on developing clothing for shielding aviation crew members from natural radiation during air travel. Materials were selected considering their radiation-shielding properties. A tungsten double-layered composite yarn and a polyethylene terephthalate (PET) fiber fabric containing BaSO_4_ were manufactured. The characteristics and shielding performances of the products were analyzed. Prototypes of a protective scarf (for shielding the thyroid gland) and apron (for shielding the torso) for flight attendants were produced. A lightweight fabric was produced that neither restricts the movement of the wearer nor causes them skin discomfort. The shielding performances of the tungsten composite and PET fiber fabrics containing BaSO_4_ were 0.018 mmPb and 0.03 mmPb, respectively, demonstrating low-dose shielding that may be useful for protecting aviation crew members from scattered rays. The characteristics of the developed fibers are comparable to those of materials used in clothing production; therefore, low-dose radiation-shielding clothing could be manufactured for use in aviation, medical, and other industries.

## Introduction

Natural cosmic radiation generated in space travels through the planet’s atmosphere to reach the surface of the earth. While most cosmic radiation is blocked by the earth’s magnetic field in the Van Allen radiation belts^1^, cosmic radiation is difficult to control artificially. Therefore, the human population is continuously affected by cosmic radiation reaching the surface of the earth. According to previous literature published by the International Commission on Radiological Protection (ICRP), the average exposure of the general public to natural radiation is 2.4 to 3 mSv per year^2^, but as natural radiation cannot be controlled, it is not considered during exposure dose management. However, when natural radiation increases due to human activities, it is considered when formulating radiation protection measures. For example, such measures are considered for miners working underground for extended periods and may be exposed to crustal radiation. Further, aviation crews that fly at high altitudes are exposed to elevated cosmic radiation levels, which are exacerbated by long cumulative flight times. Such exposure should therefore be considered during radiation safety management^3^. Generally, low-dose radiation refers to a radiation dose below 100 mSv. Most of the radiation exposure of the general public occurs in this range, either from natural or medical exposures. Therefore, the risk of low-dose radiation must be properly understood. While some researchers argue that epidemiological studies have yet to directly link the increased incidence of cancer with low-dose radiation^4,5^, others (including the International Cancer Institute) claim that low-dose radiation is globally recognized to result in an increased risk of cancer. Many agree that the linear no-threshold model for low-dose radiation is the most appropriate model for dosage response^6,7^. Therefore, precautionary measures should be implemented to protect people experiencing elevated exposure to low-dose radiation. This particularly applies to occupations that include frequent high-altitude aviation. Therefore, the effects of low-dose radiation should be considered in terms of diagnosis, treatment, testing, occupational radiation exposure, and frequency of air travel^8^.

Most public exposure to natural cosmic radiation occurs via air travel; the higher the plane's altitude, the closer it is to the source of cosmic radiation, and therefore, the greater the risk. In general, for aviation crew members working at high altitudes, the average annual effective exposure dose is estimated to be in the range of 3 to 5 mSv, and the cumulative exposure dose is estimated to be approximately 75 mSv when estimated for the total working period before retirement^9,10^. In particular, for aviation crew members using polar routes, the effective exposure dose is estimated to be approximately 6 to 7 mSv^11,12^. Considering that the average annual radiation exposure dose for workers at nuclear power plants is less than or equal to 2 mSv, the radiation exposure of aviation crew members is comparatively high considering other occupations, and being a crew member can be considered an a high-risk occupation^13^. Regarding medical radiation, a chest X-ray provides a dosage of 0.03 to 0.05 mSv, which is lower than the annual exposure dose of aviation crews, and a brain computerized tomography scan is 5 to 8 mSv, which is similar to the annual exposure dose of aviation crews^14^.

Previous literature has shown radiation to be a major cause of thyroid cancer. According to many studies, people who have undergone radiation therapy, survivors of atomic bomb explosions, and residents of areas near nuclear accidents have reported an increase in the incidence of thyroid cancer^15,16^. Although there are no significant statistics on the prevalence of thyroid cancer resulting from exposure to low-dose radiation, this radiation can be considered unsafe because it increases the risk of a population group^17^. Therefore, as with nuclear power plant workers, aviation crew members are exposed to an increased risk of thyroid cancer. Previously collected data has shown that thyroid cancer has a higher incidence in women than in men compared to other types of cancer; further, more women than men tend to be flight attendants. Depending on the duties of aviation crew members, their exposure dose to cosmic radiation is higher than that of the general public. Therefore, active exposure management in this industry is necessary^18,19^.

Medical institutions recommend lead shields with an equivalent of 0.25 to 0.5 mmPb as the standard for shielding from small doses of radiation for staff that are particularly exposed to radiation. Such staff generally wear shielding clothing to protect themselves from scattered rays. These rays are the result of indirect rather than direct radiation^19,20^. However, for aviation crew members, the weight of such shielding suits hampers their usability. Medical workers use a composite sheet of lead and rubber in their aprons; however, due to the dangers associated with lead, composites with eco-friendly materials such as tungsten, barium sulfate (BaSO_4)_, and bismuth have been used in recent years^21^.

When shielding fibers are used for radiation-shielding purposes, their performance tends to decline rapidly owing to the presence of fine pinholes between the fiber tissues. Therefore, in this study, we proposed a high-density weaving method that uses barium-sulfate-containing polyethylene terephthalate (PET) fibers and developed a composite yarn in which these fibers are doubly covered on a tungsten wire. We attempted to develop a woven fabric in a natural shape that will not cause friction-related damage, such as that which may occur on the skin. In addition, upon the commercialization of this material for clothing and other purposes, it should be lightweight and not interfere with the crew’s work. Crew uniforms, including aprons and scarves, made of this material should be easy for the flight crew to use.

## Methods

**Figure 1:**
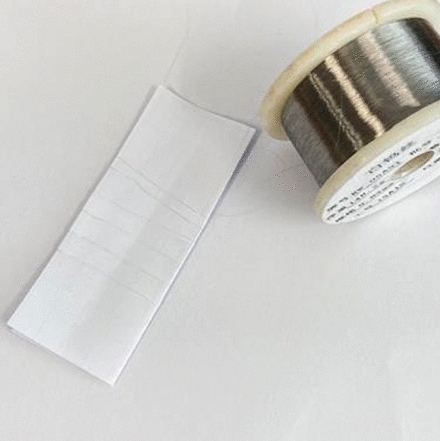
Tungsten wire (50 μm) used in the manufacture of the shielding fabric.

**Figure 2:**
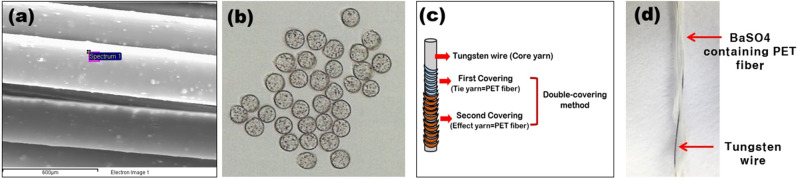
(**a**) Side-view scanning electron micrography (SEM) image of PET fiber yarn containing BaSO_4_; (**b**) cross-section microscopic image; (**c**) schematic diagram of composite yarn forming a double layer on the BaSO_4_-containing PET yarn on a tungsten wire; (**d**) SEM image of the manufactured tungsten composite yarn.

Tungsten was selected for the purposes of radiation shielding. It delivers a similar shielding performance to that provided by lead, but it is lighter and can maintain its strength. Tungsten is widely used as an alternative material to lead because of its similar shielding performance; its atomic number is 74, atomic weight is 183.84 g/mol, and density is 19.25 g/cm^3^^22^. As shown in Fig. 1, a tungsten wire with a maximum diameter of 50 µm was first obtained and used as the core yarn of a composite yarn. When tungsten wire is woven directly into a fabric, there is a risk of the wire being cut during weaving owing to the high tensile strength of tungsten; this is particularly an issue when manufacturing products using pure tungsten metal fabrics. Further, pinhole gaps between the warp and weft fibers can form during the weaving process, which may reduce the radiation-shielding performance of the resulting material. We therefore increased the density of the synthesized fabric by blending another shielding substance into the fabric. This was done by applying a double layer of PET fiber containing 5% BaSO_4_ onto the tungsten wire (Fig. 2). Similar to tungsten, BaSO_4_ is also an eco-friendly material that can replace lead for providing radiation protection. The density of barium sulfate is 4.499 g/cm^3^ and its molecular weight is 233.43 g/mol; it was selected based on its low health risks and suitability for use in clothing fabrics^23^.

**Figure 3:**
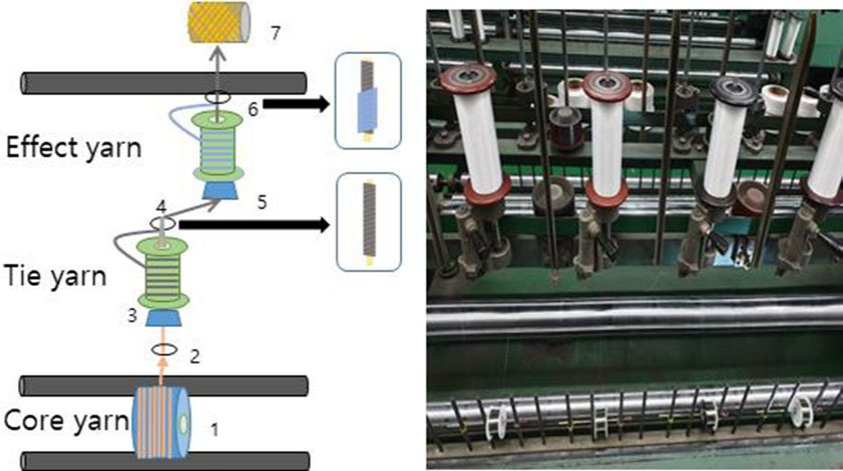
Manufacturing of the double-layered tungsten wire composite yarn using PET fiber containing BaSO_4_.

The PET fiber used in the PET shielding fabric was prepared by first nanosizing BaSO_4_ into 400-nm nanoparticles. These nanoparticles were then added directly to the PET substrate. The substrate then underwent a tempering procedure involving a melt-spinning process (Fig. 2). Figure 3 shows the process of developing a double covering on the tungsten wire using the PET fiber containing BaSO_4_. The resulting material was manufactured using tungsten wire with a double-layer covering of PET fiber as the core yarn. The double covering of the PET fibers onto the tungsten wires served to reduce the coldness of the tungsten wires upon contact with the skin. This material structure also increases the flexibility of the fiber, while improving both the radiation-shielding efficiency and weaving process.

**Figure 4:**
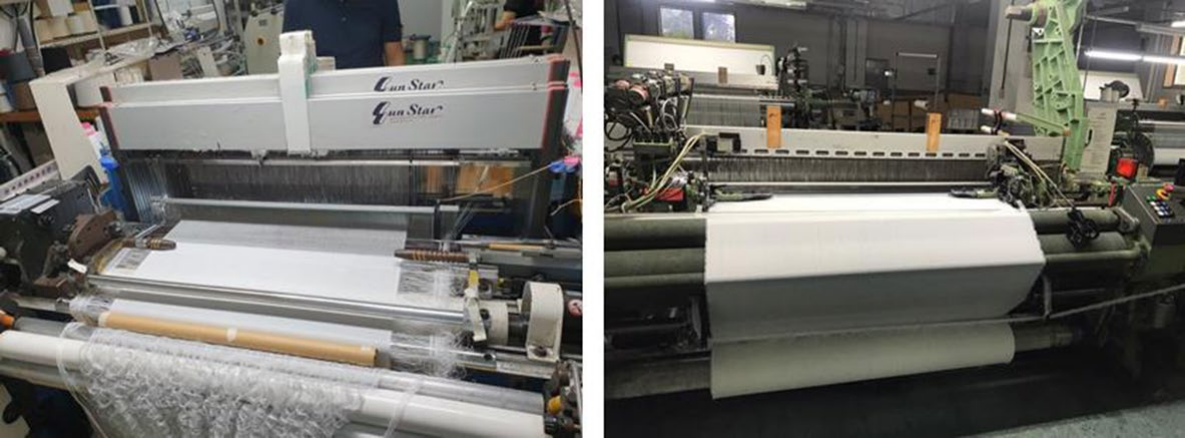
Shielding fabric weaving process conducted using an HTVS 8/S20 machine.

Using a composite based on tungsten and BaSO_4_-containing PET fibers, the fabric shown in Fig. 4 was woven and used to make a prototype apron. A second form of this fabric was also produced to enable the analysis of the weaving process and the shielding performance of the resulting material. The second form of this fiber was woven using only BaSO_4_-containing PET fibers, and tungsten was excluded. The tungsten-based composite yarn fabric was used to make a prototype apron due to its superior physical properties (high density and rigidity), while the fabric containing only BaSO_4_ was used to produce a prototype scarf based on the fabric’s flexibility and relatively soft texture. Therefore, clothing was produced that could shield areas of the body that are highly sensitive to radiation, such as the thymus gland, thyroid glands, and genitals.

As radiation penetrates the shielding materials, the radiation dose and energy are reduced via interactions with these materials. These methods of measuring radiation dose can be used to calculate the rate of loss of the original energy levels, or to measure the strength before and after transmission over distance. In this study, we analyzed the reduction of energy generated at a certain distance. Therefore, the scattered rays from an indirect X-ray (that is, a low-dose area) were considered, and radiation shielding was designed by varying the original energy intensity with respect to the energy absorption range per unit area^24^.

1 $$\mathrm{I}\left(d\right)={I}_{0}{\mathfrak{e}}{\sum }_{\beta }d, {N}_{\alpha }=\beta $$

Equation (1) expresses the radiation-shielding effects of the manufactured shield fiber, where ${\mathrm{I}}_{0}$ denotes the strength of the dose transmitted through the shield fiber and $d$ denotes the thickness of the shield fiber. $\upbeta$ denotes the total area of the substance contained in the reduced fiber that acts as a radiation shield, and this area is the same as the unit area ${\mathrm{N}}_{\alpha }$ that absorbs the generated low dose of radiation. $\mathrm{N}$ is the number density (number of atoms/mm^3^) of the shielding material and $\mathrm{\alpha }$ is the radiation micro-absorption cross-sectional area (mm^3^). Therefore, ${\sum }_{\beta }$ can be described in this study as the low-dose radiation absorption cross-sectional area (mm^3^) to be shielded. To reduce the thickness of the shielding fibers and increase ${\mathrm{N}}_{\alpha }$, this method required the selection of a material with a superior shielding performance, such as tungsten. Further, the cross-sectional area for radiation micro-absorbing may be increased^24^.

Therefore, in this study, shielding fibers were used to manufacture a soft cloth to demonstrate the effectiveness of several overlayed layers; this cloth would be used to make scarves for shielding the neck (including the thyroid glands) of flight attendants. The radiation was energy-attenuated as it passed through the fiber. The intensity of radiation energy varied with its distance and direction with regard to the subject. Considering the thickness and density of the shielding fiber through which the radiation energy was able to pass, the energy intensity was determined via Eq. (2)^25^. The radiation energy becomes attenuated while passing through the shielding fiber medium due to interactions with the shielding material. Therefore, increasing both the thickness and density of the medium is effective for achieving energy attenuation.

2 $$\mathrm{I}={\mathrm{I}}_{0}e^{-\mu \chi }$$

$\mathrm{I}$: Energy intensity that has passed through the shield fiber.

$${\mathrm{I}}_{0}$$: Transmittance strength.

$\upmu$: Linear attenuation coefficient.

$\upchi$: Shielding fiber thickness (μm).

3 $$\mathrm{X}-\mathrm{ray\,shielding\, rate }\,(\mathrm{\%})=(1-\frac{\kappa }{{\kappa }_{0}})\times 100$$

In Eq. (3), ${\kappa }_{0}$ denotes the incident dose (mR) and $\upkappa$ is the transmitted dose (mR).

The standard shielding performance of the shielding fiber was evaluated for equivalent shielding effectiveness based on lead. Therefore, the shielding performance was presented based on the lead equivalent. Lead equivalent is expressed in units of mmPb, and is presented as Eq. (4). The lead equivalent ${F}_{Pb}$ of the shielding fiber can be expressed as the correlation between the thickness *d* of the shielding fiber and the linear attenuation coefficient ${\mu }_{F}$ of tungsten and barium sulfate and the linear attenuation coefficient ${\mu }_{Pb}$ of lead.

4 $${F}_{Pb}=\frac{{\mu }_{F}}{{\mu }_{Pb}}\times d$$

**Figure 5:**
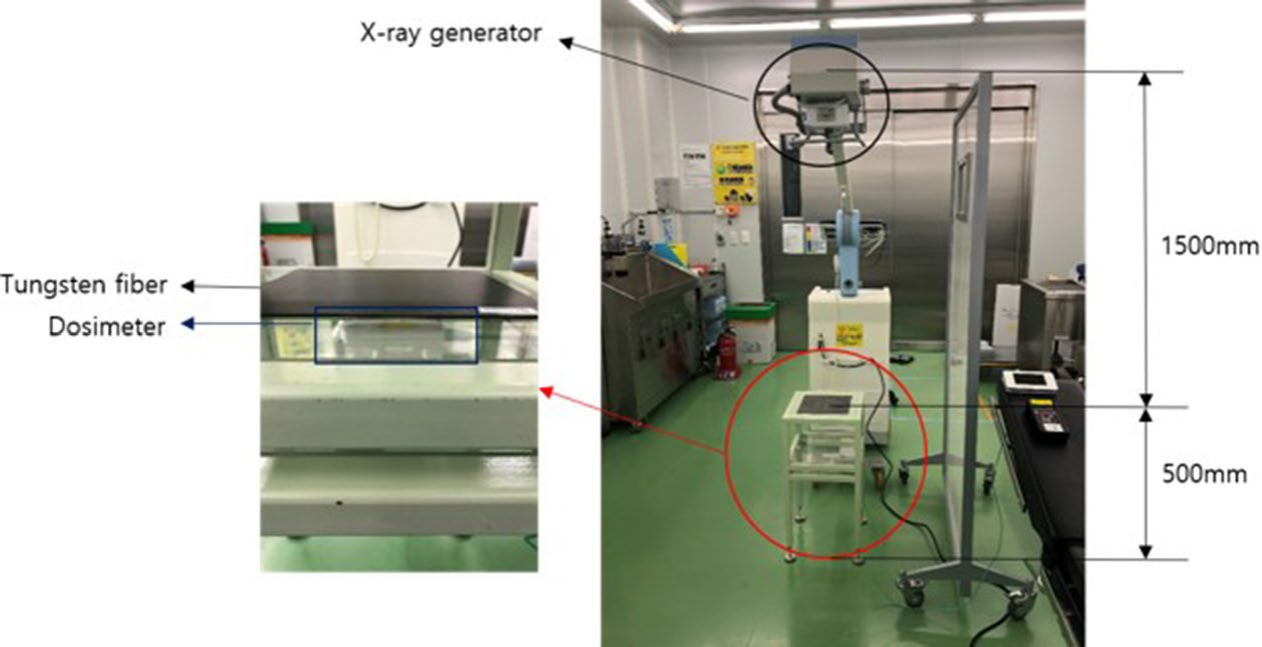
MOBIX-1000 and Listem, 2010 X-ray equipment used for the evaluation of the radiation shielding provided by the shielding fibers of the tungsten composite fabric and BaSO_4_ fabric.

To evaluate the shielding performances of the tungsten composite fabric and BaSO_4_ fabric, the shielding performance by energy zone was evaluated using medical X-ray-generating devices (MOBIX-1000, Listem, 2010) (Fig. 5).

**Figure 6:**
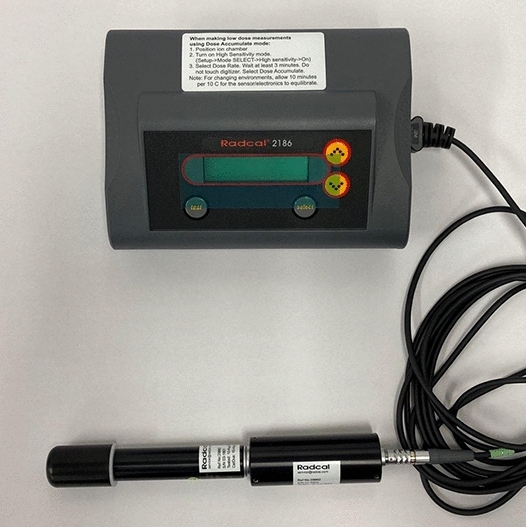
Low-dose radiation measuring equipment (Radcal 2186 [Accu-Dose], Radcal Co, 2017, Correction 2020).

The measurement method that was utilized was applied in compliance with that utilized by the lead equivalent test method (KS A 4025: 2017)^26^. An ion chamber meter device was used for radiation measurements (Radcal 2186 [Accu-Dose], Radcal Co, 2017, Correction 2020) (Fig. 6).

## Results

**Figure 7:**
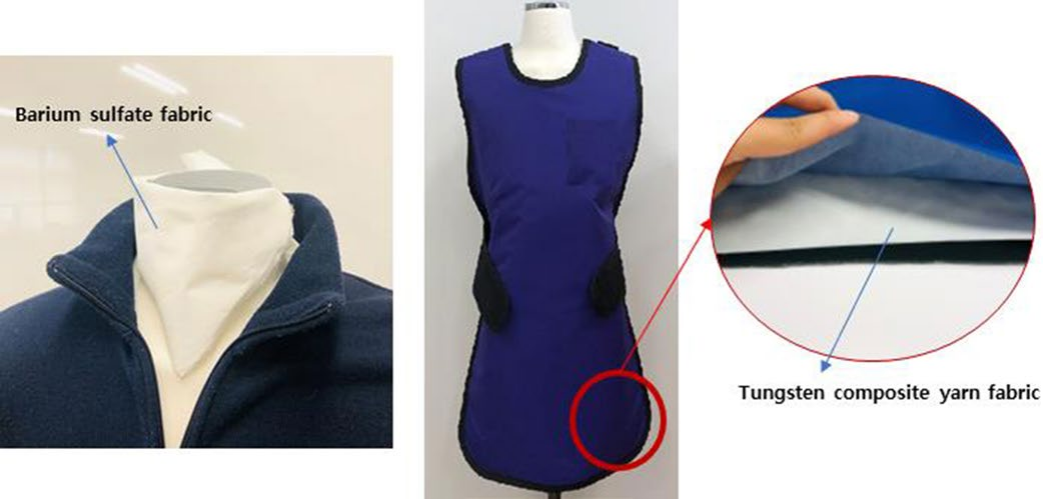
Prototype radiation shield clothing: (**a**) scarf made from PET containing BaSO_4_ and (**b**) (with inset) apron and radiation shield clothing items manufactured using the tungsten composite fabric.

Table 1 shows the characteristics of the PET fibers containing BaSO_4_. It was ensured that the produced cloth material exhibits a soft texture such that it could be worn against the skin as a scarf for protection of the thyroid gland from radiation (Fig. 7). The fabricated material was developed as 75D34F (i.e., 34 filaments representing 75 deniers) with a tensile strength of 3.14 gd, determined via a maximum stretching process. The maximum strength and flexibility of the fibers was achieved, which was enhanced through the use of multiple layers (Table 1).

**Table 1:** Characteristics of a PET fiber containing barium sulfate.

PET yarn (BaSO_4_)	SEM: side view (× 400)	SEM: cross-section (× 600)	Fineness (D)	Tensile strength (g/d)	Elongation at break (%)
			75.1	3.14	29.4

**Figure 8:**
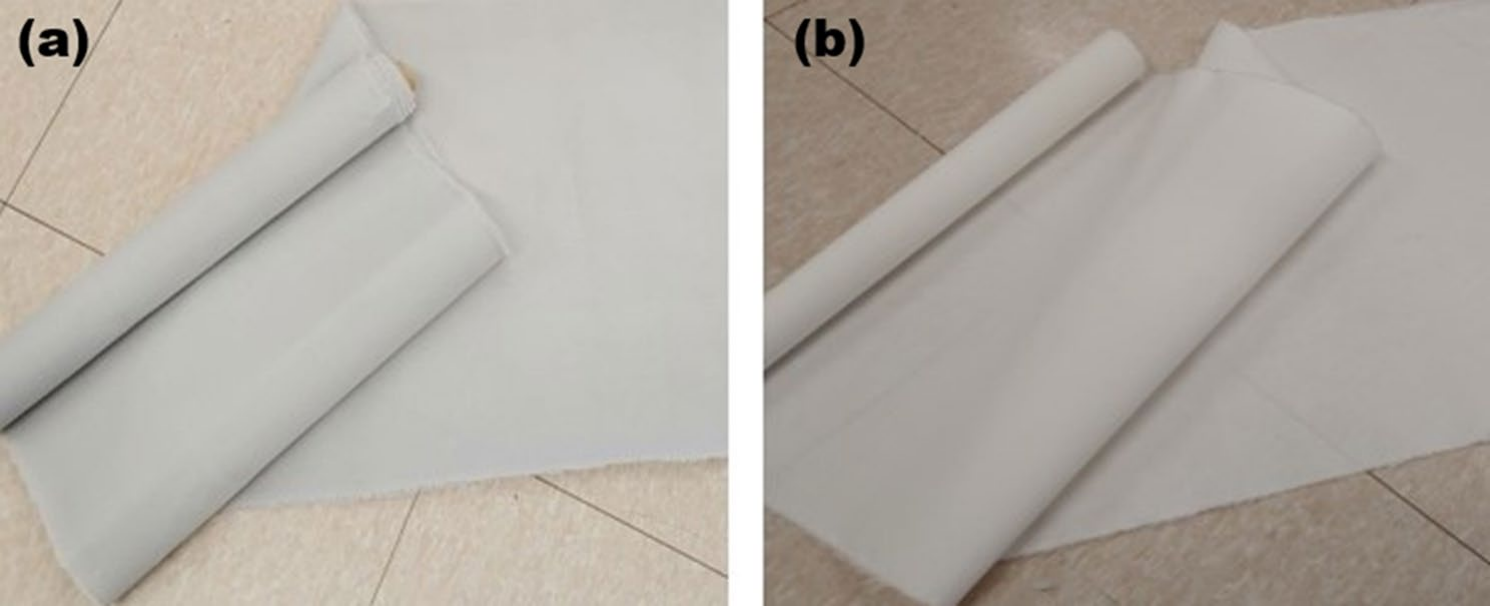
Woven radiation-shielding fibers: (**a**) fabric using tungsten composite yarn and (**b**) fabric using PET fiber containing BaSO_4_.

Table 2 shows the characteristics of composite yarns containing tungsten and BaSO_4_. The structure of the BaSO_4_-containing PET yarn that was doubly layered onto the tungsten wire and formed the core yarn (i.e., the composite yarn) was produced by twisting at 300 T/M (Twists/meter). This was implemented to attain both the strength and flexibility required to make the fabricated items suitable for use in a protective apron. The two fabrics were similar in appearance, and for the purpose of comparison, the yarn was maintained in its undyed form (Fig. 8).

**Table 2:** Characteristics of composite yarn with a double covering of polyester (PET) fiber containing barium sulfate on a tungsten wire core.

Conjugate yarn (BaSO_4_ and W)	SEM: side view (× 400)	SEM: cross-section (× 600)	Fineness (D)^a^	Tensile strength (g/d)	Elongation at break (%)	Diameter of tungsten core yarn	BaSO_4_ effect yarn & tie yarn
			492.9	1.59	4.4	50 μm	75/36 (300 TM)^b^

^a^Denier (D): the unit of thickness of fiber, with 1D being 9,000 m of thread weighing 1 g.

^b^TM (Twist/meter): the number of twists given to a 1 m thread.

Table 3 shows the characteristics of the fabric woven for radiation shielding. The thicknesses of the fabric woven using PET yarn containing BaSO_4_, and that woven with tungsten-based composite yarn, were determined to range from 0.19 to 0.20 mm and 0.25 to 0.31 mm, respectively. The weight and tensile strength of the tungsten-based composite yarn fabric were superior. In contrast, the density of the fabric woven with the tungsten-based composite yarn showed inferior quality because tungsten wire has low tensile elongation properties. Moreover, the number of warp and weft yarn counts woven per unit area was limited when using this material. For this reason, this fiber is somewhat lacking in terms of softness.

**Table 3:** Properties of the produced fabrics.

Class	Thread count (thread/inch)		Tensile strength (N)	Elongation at break (%)	Tearing strength (N)	Weight (g/m^2^)	Thickness (mm)
	Warp	Weft					
PET yarn (BaSO_4_)	200	100	(warp) 740 (weft) 290	(warp) 34 (weft) 20	(warp) 16 (weft) 9	114–118	0.19–0.20
Conjugate yarn (BaSO_4_ and W)	80	60	(warp) 600 (weft) 420	(warp) 7.6 (weft) 3.3	(warp) 58 (weft) 113	289–300	0.25–0.31

The results shown in Table 4 were derived from the evaluation of the radiation-shielding performances of the manufactured fabrics. These fabrics were found to provide relatively effective low-energy shielding, and the tungsten composite yarn fabric provided a better shielding performance than the BaSO_4_ fabric. In the standard lead equivalent comparison experiment, the tungsten composite yarn fabric was found to be 0.03 mmPb, while the BaSO_4_-containing PET yarn had a particularly low lead equivalent, as well as a markedly low shielding rate. The results of this experiment were obtained using single-sheet fabrics.

**Table 4:** Shielding performance evaluation results of the barium sulfate-based fabric and tungsten composite yarn-based fabric.

Radiation type	Effective X-ray Energy (keV)	Shielding rate (%)		Lead equivalent (mmPb)	
		BaSO_4_ fabric	W Composite yarn fabric	BaSO_4_ fabric	W Composite yarn fabric
X-ray	27.7	72.7	83.9	0.018	0.03
	29.9	57.9	72.0		
	33.6	48.7	63.5		
	46.7	41.2	57.4		
	52.3	37.5	50.4		
	59.7	33.1	45.2		

## Discussion

The natural cosmic radiation exposure that occurs during high-altitude aviation corresponds to the low doses of radiation used by medical institutions as the scattering lines are similar. No matter how small the radiation dose, there is theoretical risk that is proportional to the dose value; a dose as low as 1 mSv can result in an increased risk of cancer. Cosmic radiation is a low-dose radiation, and the theoretical risk it can cause cannot be ignored. However, the risk resulting from low doses of radiation is small compared to many other cancer risk factors. Nevertheless, patients and medical staff have been proactive about procuring protection from low-dose scattered radiation^27–29^.

Radiation exposure in medical institutions mainly occurs from indirect rays, which are scattered rays with a radiation dose of less than 100 mSv. The shielding associated with protection from such rays is currently being changed from lead-centered shielding to shielding created using eco-friendly lightweight materials. These eco-friendly materials, such as barium sulfate, bismuth oxide, and tungsten, have been commercialized as radiation-shielding materials that can replace lead, which is a heavy metal. They are mainly used to manufacture sheets and films that are used as shielding materials in medical institutions (e.g., airfron)^30,31^.

In some radiation shielding experiments, there is also research on radiation reducing fibers. These fibers may be commercialized by applying a liquid shielding material to a nonwoven fabric. However, the uniformity and reproducibility of the shielding performance, which are the core factors in shielding, cannot be guaranteed when using this method^32^. Therefore, it is necessary to obtain a uniform dispersion of the shielding material. The dispersibility of shielding materials is highly influenced by the material mixture, and there is therefore a limit when attempting to achieve a uniform dispersion during manufacturing^33^. It is difficult to maintain a consistent shielding performance even if the same shielding material is utilized because the dispersion of the shielding material is affected by both the polymer material and mixing process, as well as by the conditions of the manufacturing process^34^. The pressure conditions, coating method, and temperature can significantly affect the structural conditions of the internal substances. Therefore, in recent years, single-material shielding films have garnered significant interest, and tungsten shielding paper has been reported upon as a representative product of such materials^35^.

As shown in this study, the issue of uneven coatings can be partially solved via weaving with fibers. By weaving using a composite yarn in which fibers containing barium sulfate are double-covered onto a single-material tungsten wire, the dispersion conditions of the shielding substances that affect the physical properties of the resulting material are minimized and the reproducibility of the shielding performance can be maintained. However, there is a disadvantage in that the shielding effects are reduced due to fine pinholes that are created when weaving with fibers. Therefore, as was done in this experiment, fabrics may be produced via a double-layer stacking method to increase shielding from direct X-rays while weaving at high density. Additionally, dispersing BaSO_4_ nanoparticles onto the fabric may be considered.

Natural cosmic radiation penetrates the atmosphere from space. As the atmosphere becomes increasingly thin with increasing altitude, the upper atmosphere is subjected to greater levels of radiation; hence, the higher the altitude of an aircraft, the greater the radiation levels in the aircraft interior (0.01 mSv per annum per 30 cm rise above sea level). The effective dose limit for workers subjected to radiation is stipulated to not exceed 100 mSv over 5 years and remain within 50 mSv per annum^36,37^. The amount of exposure of aviation crew members to ionizing radiation due to natural cosmic radiation is determined by such factors as the type of flight, flight time, flight altitude, and flight latitude. Civil aircrafts are is generally exposed to radiation with an average range of 2.0 to 5.2 μSv/h depending on the route and method of measurement^38^. Therefore, the relatively high dose of radiation exposure of aviation crews is cumulative and can adversely affect the health of crew members. Moreover, studies have determined that the cancer incidence in aviation crew members is higher than that in the general population^39,40^.

Therefore, active efforts are required to protect flight attendants from radiation, and it must be possible to shield at least 0.07 to 0.10 μSv/h. The Lightweight flexible clothing manufactured from shielding with lead equal to 0.03 mmPb as suggested in this study has a shielding rate of at least 50% at 52.3 keV of effective X-ray energy in high-energy medical radiation areas. Hence, effective shielding is possible for flight attendants; further, unlike cumbersome radiation protection devices, the proposed clothing does not interfere with the routines and functions of the attendants.

Furthermore, in radiation-shielding areas in medical facilities, the areas affected by scattered rays are larger than the direct radiation-shielding areas. However, it is uncomfortable to wear shielding clothes that weigh approximately 3 kg for long periods. Therefore, it is possible to provide shielding in the scattered ray areas by using clothes made of lightweight flexible shielding fabric, such as those presented in this study.

In summary, the present study was conducted based on the need for radiation-shielding fibers in the manufacturing industry to enable the production of a range of fabric products that can reduce the cosmic radiation exposure of aviation crew members, as well as potentially providing radiation shielding for medical staff.

## Conclusion

This study demonstrated that clothing manufactured using tungsten composite fabric and PET fiber fabric containing barium sulfate can provide shielding from low-dose radiation with 0.018 mmPb and 0.03 mmPb shielding performances, respectively. The manufactured clothing is lightweight and does not interfere with work-related activities. Such clothing is of significant importance because air crews flying at high altitudes are exposed to elevated levels of natural cosmic radiation. This clothing may also find use in other industries, such as the medical and mining industries, where workers are exposed to on-site radiation. The double-covering yarn manufacturing technology developed in this study may therefore be applied to the development of other industry-specific shielding materials, which could be further used to manufacture customized shielding clothing. The subsequent commercialization of such fabrics would enable the manufacturing of a variety of clothing items and improve the safety conditions of workers in various industries.
